# The economic costs of alcohol consumption in Lithuania, 2015–20

**DOI:** 10.1093/eurpub/ckaf069

**Published:** 2025-05-09

**Authors:** Vaida Liutkutė-Gumarov, Claire de Oliveira, Auksė Domeikienė, Lukas Galkus, Ahmed S Hassan, Shannon Lange, Laura Miščikienė, Birutė Peištarė, Janina Petkevičienė, Ričardas Radišauskas, Jürgen Rehm, Pol Rovira, Ilona Tamutienė, Mark James Thompson, Mindaugas Štelemėkas

**Affiliations:** Health Research Institute, Faculty of Public Health, Lithuanian University of Health Sciences, Kaunas, Lithuania; Centre for Addiction and Mental Health, Institute for Mental Health Policy Research, Toronto, ON, Canada; Dalla Lana School of Public Health, University of Toronto, Toronto, ON, Canada; Campbell Family Mental Health Research Institute, Centre for Addiction and Mental Health, Toronto, ON, Canada; Department of Preventive Medicine, Faculty of Public Health, Lithuanian University of Health Sciences, Kaunas, Lithuania; Health Research Institute, Faculty of Public Health, Lithuanian University of Health Sciences, Kaunas, Lithuania; Centre for Addiction and Mental Health, Institute for Mental Health Policy Research, Toronto, ON, Canada; Dalla Lana School of Public Health, University of Toronto, Toronto, ON, Canada; Centre for Addiction and Mental Health, Institute for Mental Health Policy Research, Toronto, ON, Canada; Campbell Family Mental Health Research Institute, Centre for Addiction and Mental Health, Toronto, ON, Canada; Department of Psychiatry, Faculty of Medicine, University of Toronto, Toronto, ON, Canada; Faculty of Medicine, Institute of Medical Science, University of Toronto, Toronto, ON, Canada; Health Research Institute, Faculty of Public Health, Lithuanian University of Health Sciences, Kaunas, Lithuania; Department of Public Administration, Faculty of Political Science and Diplomacy, Vytautas Magnus University, Kaunas, Lithuania; Health Research Institute, Faculty of Public Health, Lithuanian University of Health Sciences, Kaunas, Lithuania; Department of Preventive Medicine, Faculty of Public Health, Lithuanian University of Health Sciences, Kaunas, Lithuania; Department of Environmental and Occupational Medicine, Faculty of Public Health, Lithuanian University of Health Sciences, Kaunas, Lithuania; Institute of Cardiology, Lithuanian University of Health Sciences, Kaunas, Lithuania; Centre for Addiction and Mental Health, Institute for Mental Health Policy Research, Toronto, ON, Canada; Dalla Lana School of Public Health, University of Toronto, Toronto, ON, Canada; Campbell Family Mental Health Research Institute, Centre for Addiction and Mental Health, Toronto, ON, Canada; Department of Psychiatry, Faculty of Medicine, University of Toronto, Toronto, ON, Canada; Faculty of Medicine, Institute of Medical Science, University of Toronto, Toronto, ON, Canada; PAHO/WHO Collaborating Centre, Centre for Addiction and Mental Health, Toronto, ON, Canada; Center for Interdisciplinary Addiction Research (ZIS), Department of Psychiatry and Psychotherapy, University Medical Center Hamburg-Eppendorf (UKE), Hamburg, Germany; Program on Substance Abuse & WHO European Region Collaboration Centre, Public Health Agency of Catalonia, Barcelona, Catalonia, Spain; Program on Substance Abuse & WHO European Region Collaboration Centre, Public Health Agency of Catalonia, Barcelona, Catalonia, Spain; Health Research Institute, Faculty of Public Health, Lithuanian University of Health Sciences, Kaunas, Lithuania; Department of Public Administration, Faculty of Political Science and Diplomacy, Vytautas Magnus University, Kaunas, Lithuania; Vytautas Kavolis Transdisciplinary Institute for Social Sciences and Humanities, Vytautas Magnus University, Kaunas, Lithuania; Health Research Institute, Faculty of Public Health, Lithuanian University of Health Sciences, Kaunas, Lithuania; Institute for Wealth and Asset Management, Zurich University of Applied Sciences, Winterthur, Switzerland; Health Research Institute, Faculty of Public Health, Lithuanian University of Health Sciences, Kaunas, Lithuania; Department of Preventive Medicine, Faculty of Public Health, Lithuanian University of Health Sciences, Kaunas, Lithuania

## Abstract

Alcohol *per capita* consumption in Lithuania among the population 15 years of age and older has been among the highest globally in recent decades. Long-term alcohol consumption trends and drinking patterns signal a significant public health problem, as well as social and economic losses. This study aimed to estimate the economic burden associated with alcohol consumption in Lithuania from 2015 to 2020. We used a cost-of-illness methodology with the human capital approach to estimate the economic burden and applied a prevalence-based approach. Using multiyear data, we estimated both, direct and indirect costs. Direct costs included healthcare and childcare, law enforcement, and justice system costs. Indirect costs included costs of productivity loss due to premature mortality. The total economic cost of alcohol consumption in Lithuania between 2015 and 2020 was estimated at an annual average of €542.958 million (in 2020 Euros) or about 1.18% of the Lithuanian total Gross Domestic Product. The highest proportion (65%) of the estimated costs was associated with productivity losses due to premature mortality. Alcohol use places a considerable burden on Lithuanian society in terms of illness, injury, death, and economic costs. Alcohol control policies, in particular excise taxation increases and availability restrictions have been shown to decrease this burden.

## Introduction

Alcohol use remains one of the leading risk factors for infectious and noncommunicable diseases and injuries worldwide, as well as a key cause of many societal problems. This altogether translates into negative effects on longevity, employment, and productivity. A 2009 global burden of disease and injury review reported economic costs attributable to alcohol use and alcohol use disorders in high-income countries to be as high as 2.5% of their respective Gross Domestic Product (GDP) [1]. The most recent systematic review confirms that alcohol consumption led to substantial costs globally with an equivalent of 1.5%–2.6% of the respective country’s GDP, the latter figure reflecting a situation where all cost components had been included [2].

The health and economic burden for individuals and society is highest in countries with the highest alcohol consumption and most harmful drinking patterns. This is particularly relevant in Lithuania, where consumption of alcohol is higher than in many other European countries, binge drinking is widespread, and alcohol-attributable mortality is among the highest in the European Union [3, 4].

Since 1993, the year with the lowest level of alcohol consumption after regaining independence, alcohol consumption in Lithuania has more than doubled, starting at 6.2 litres of pure alcohol per capita and reaching its peak in 2011–12 at more than 15 litres per capita [5]. Consequently, Lithuanian policymakers have responded to the problem of alcohol harm with strong alcohol control policies, primarily implemented between 2008 and 2018 [5]. While alcohol-attributable mortality remains high in Lithuania, there is a positive trend, predominantly amongst males, associated with the implementation of the World Health Organization’s (WHO’s) ‘best buys’ [3].

Timely, comprehensive cost estimates can inform important healthcare and public health decisions, including better allocation of public funds and prevention budgets and understanding the efficacy of policy interventions. In the case of Lithuania, where the Law on Alcohol Control was one of the most frequently amended acts in Lithuania between 1995 and 2020 [6], such estimates can also help to keep existing policies in place for a longer period or accelerate the strengthening of the law. Furthermore, cost estimates that account for the costs to non-drinkers can help raise public awareness of the spillover effects of the burden of alcohol use. The estimation of the alcohol-attributable economic costs in high-income and high alcohol consumption settings adds to the existing evidence to demonstrate that alcohol use continues to harm societies across different regions.

This study aims to measure the direct and indirect economic costs of alcohol consumption in Lithuania in the 2015–20 period.

## Methods

### Study design

To estimate the economic costs of alcohol, we used a prevalence-based cost-of-illness approach [7]. We estimated both direct and indirect costs, including four major cost components—healthcare, childcare, law enforcement, and justice system costs—as well as the loss of productivity due to premature mortality from 2015 to 2020 (for standard categories in alcohol cost studies, see Manthey *et al.* [2]). We applied the human capital approach to estimate the costs of productivity loss due to premature mortality. All alcohol-attributable costs were adjusted to 2020 Euros based on changes in the annual consumer price index (CPI) in Lithuania (see Table S1). Additionally, all costs were converted into percentages relative to Lithuania’s GDP for international comparability [8].

### Calculation of alcohol-attributable fraction

We used disease or health-outcome-specific alcohol-attributable fraction (AAFs) to quantify the proportion of an outcome, which could have been avoided in a scenario of zero alcohol consumption. In this study, diseases were classified according to the International Statistical Classification of Diseases and Related Health Problems 10th Revision (ICD-10), and repartitioned into three major alcohol-related disease groups, as follows:

I = Fully alcohol-attributable causes

II = Partially alcohol-attributable causes

III = External causes

For the analysis of healthcare costs and the costs of lost productivity, we used population-attributable fraction methodology, which integrates alcohol exposure in the specific population (Lithuania) with corresponding relative risks (RRs) [9, 10]. We obtained alcohol consumption data from the WHO Global status report on alcohol and health and treatment of substance use disorders [11], while RRs were taken from Shield *et al.* [12] and based on a recent analysis on best-fitting sex-specific RRs for Lithuania [3]. See Table S2 for a detailed list of disease (ICD-10) categories and AAFs for each year.

For the analysis of the lost productivity costs, we considered disease group I 100% attributable to alcohol. For diagnoses partially attributable to alcohol, we applied RRs provided by Rehm *et al.* (2024); details can be found elsewhere [3]. By using the latter approach, we ensured that the RRs pulled from a large Russian retrospective case-control study were used to estimate AAFs for Lithuanian males, while global RRs were used for Lithuanian females. This methodological approach was previously recommended for former Soviet Union countries with high alcohol consumption [3]. For disease group III, we used national autopsy data on the cause of death (see Table S3 and Miščikienė *et al.* [13] for more details). For concision, Table S3 provides a 2020 snapshot of the sex- and age group-specific AAFs.

To calculate the AAFs for criminal offences attributable to alcohol use, we used total annual numbers of criminal offences and numbers of criminal offences committed by alcohol-intoxicated persons (see Table S4). We obtained data from the Criminal Offences Departmental Register of the Information Technology and Communications Department under the Ministry of the Interior of the Republic of Lithuania [14].

The analysis of data provided by the Police Department [15] enabled the estimation of the annual AAFs of road traffic accidents caused by driving under the influence of alcohol (see Table S5). These estimates encompass road traffic accidents involving cars, pedestrians, and cyclists.

### Direct costs

In this study, direct costs included healthcare costs, childcare system costs, law enforcement costs, and justice system costs.

#### Healthcare costs

Estimated healthcare costs include the following cost categories: outpatient services, active inpatient treatment, long-term care, reimbursable pharmaceuticals, and medical help equipment.

We obtained the health service provider-, ICD-10 disease-, and year-specific data for the healthcare services paid by the National Health Insurance Fund (NHIF) under the Ministry of Health. The costs that were used represent the reimbursement requirement presented by the health service provider. The estimation of alcohol-attributable healthcare costs is based on summarizing the annual costs attributable to specific ICD-10 disease groups and weighting by corresponding disease-group-specific average AAFs.

#### Childcare costs

##### Costs of child guardianship

We obtained the total annual number of children in guardianship and children leaving the guardianship (due to reaching adulthood) from the database of the Social Support for Families Information System [16, 17]. The proportion of children in the guardianship system due to parental alcohol abuse was identified from previous studies [18, 19]. Then, we multiplied the number of children in the guardianship system due to parental alcohol misuse by the costs incurred for their annual support per capita provided by the Department of Social Services Supervision under the Ministry of Social Security and Labour [20]. Estimates also include the one-time payments for children leaving the guardianship system due to reaching adulthood.

##### Costs of workforce supporting families affected by alcohol misuse

We estimated the pay of the workforce, including social workers, case managers, mobile team staff, psychologists, and addiction specialists, working with families experiencing social risk due to alcohol. The number of alcohol-attributable social risk families was obtained from the annual reports of the State Child Rights Protection and Adoption Service [17]. To estimate the annual costs of specialists working to support social risk families, we multiplied the number of specialists by their annual salary, obtained from the public employees within the Ministry of Social Protection and Labor.

##### Costs of social benefits

To estimate the alcohol-attributable costs of social benefits paid to social risk families experiencing social risk due to alcohol, we multiplied the average amount of social benefits per person (per year) [21] by the number of socially at-risk families misusing alcohol [16].

#### Law enforcement and justice system costs

##### Imprisonment costs

The types of crimes included in imprisonment costs were crimes against property (i.e. robberies, theft, extorsion), violent crimes (i.e. homicide, severe health impairment, and rape), and violations of public order. The costs of imprisonment were calculated by multiplying the annual number of alcohol-attributable imprisonments by the daily cost of housing for an individual in a correctional facility. The daily costs were estimated based on data obtained from the annual reports of the Lithuanian Prison Services [22]. These estimates considered the average length of imprisonment and the number of newly convicted individuals, as well as the average daily cost of maintaining a person in detention facilities, which ranged from €18.6 in 2015 to €37.3 in 2020.

##### Court costs

The alcohol-attributable costs of judicial proceedings were calculated by applying the year-specific AAF for criminal offences committed by alcohol-intoxicated individuals to the year-specific state budget allocated to the court system. The budget allocations for the court system included costs for wages and asset acquisition and ranged from €61.68 million in 2015 to €83.44 million in 2020 [23].

##### Pre-trial investigation costs

To calculate the annual cost of pre-trial investigations into criminal offences committed by alcohol-intoxicated persons, we multiplied the average cost of one pre-trial investigation by the number of criminal offences suspected to be committed by alcohol-intoxicated persons.

We used the ‘Methodology for Calculating the Cost of the Pre-Trial Investigation’ [24] approved by the Deputy Prosecutor General on 4 April 2017 to estimate the average cost of one pre-trial investigation. Based on the referenced methodology, we considered the year-specific average hourly wage of police investigators and prosecutors, their working time and retention rates. The average costs of one pre-investigation were €912, €978, €1022, €1071 and €1509, respectively, in 2015–20.

##### Traffic accidents

The cost of property damage due to road traffic accidents caused by driving under the influence of alcohol was estimated by multiplying the total annual monetary value of insurance benefits payments by the proportion of road traffic accidents attributable to alcohol, obtained from the Police Department [15]. The Bank of Lithuania provides year-specific data on the insurance benefits paid by insurance companies to those covered by motor vehicle managers civil liability and comprehensive insurance. The paid insurance benefits ranged from €157.43 million in 2015 to €250.48 million in 2019 [25].

##### Financial loss due to criminal offences

The analysis of alcohol-attributable property damage costs due to criminal offences include the number of crimes charged under 22 different articles of the Criminal Code of the Republic of Lithuania, including but not limited to crimes such as homicide, manslaughter, severe health impairment, rape, sexual assault, violation of public order, false report about a danger threatening the community or disaster, desecration of a grave or another place of public respect, and other. We obtained the total annual number of criminal offences, the number of offences committed by alcohol-intoxicated persons, and the monetary loss caused to the individuals, the state, or legal entities from the Criminal Offences Departmental Register of the Information Technology and Communications Department under the Ministry of the Interior of the Republic of Lithuania. Total monetary loss ranged from €33.48 in 2020 to €144.27 million in 2017. On average more than half (54.8%) of all murders and more than half (52.9%) of all severe health impairments committed between 2015 and 2020, were committed by a person under the influence of alcohol [26].

### Indirect costs

#### Cost of productivity loss due to premature mortality

To estimate the number of alcohol-attributable deaths for each disease in each disease group (I, II, and III), we aggregated the annual number of deaths from individual-level records obtained from the Institute of Hygiene, which maintains the Lithuanian mortality database [27]. Economic productivity costs attributable to alcohol were estimated by considering the average annual wages of males and females in Lithuania and estimating alcohol-attributable years of life lost (YLL) before the year of retirement at age 65. We used the Organization for Economic Co-operation and Development (OECD) wage statistics [28] to value the loss of productivity from YLL. We expressed lost income in 2020 € using the Lithuanian CPI (see Table S1).

## Results

The average annual alcohol-attributable economic cost in Lithuania between 2015 and 2020 was estimated at approximately €542.958 million adjusted to 2020 Euros, as shown in Table 1. The alcohol-attributable cost during the period of analysis was on average 1.18% of annual GDP and marked a reduction from 1.29% in 2015 to a 1.1% in 2018, increasing to 1.18% in 2020. Indirect costs outweighed the direct costs, representing on average 65.2% of the total cost. Significantly lower were the costs for law enforcement and justice system (13.5%), social and childcare (10.7%), and healthcare (10.5%).

**Table 1.** Summary of the alcohol-attributable costs in Lithuania, 2015–20 (numbers in Euros)

Costs, adjusted to 2020 € (95% CI)	2015	2016	2017	2018	2019	2020
**Direct costs**						
**Healthcare (€)**	**55 518 528 (52 396 112–62 543 011)**	**55 962 530 (51 409 535–62 541 857)**	**51 497 412 (46 955 509–57 219 122)**	**55 984 446 (52 071 417–62 978 069)**	**62 422 151 (56 938 837–69 660 356)**	**60 798 351 (56 076 349–67 748 886)**
Group I	5 813 638 (5 813 638–5 813 638)	5 979 608 (5 979 608–5 979 608)	5 202 618 (5 202 618–5 202 618)	5 582 438 (5 582 438–5 582 438)	6 091 709 (6 091 709–6 091 709)	5 351 640 (5 351 640–5 351 640)
Group II	28 011 670 (25 571 976–33 779 723)	27 958 346 (24 523 115–32 873 608)	24 818 604 (21 176 721–29 275 200)	26 453 799 (23 514 444–32 078 656)	31 600 297 (26 860 369–36 846 248)	31 406 617 (28 088 945–37 060 446)
Group III	21 693 219 (19 537 030–24 650 743)	22 024 576 (19 866 810–24 904 899)	21 476 190 (19 301 393–24 292 690)	23 948 208 (20 989 828–26 804 468)	24 730 144 (22 350 856–28 120 160)	24 040 096 (21 268 099–26 954 326)
**Childcare (€)**	**59 607 040**	**54 961 355**	**60 650 967**	**57 106 191**	**58 686 543**	**58 877 716**
Child guardianship	53 137 560	48 544 824	57 392 582	49 735 118	47 857 900	45 119 514
Social benefits	3 365 029	3 110 871	3 258 385	3 632 236	4 217 248	4 723 450
Workforce	3 104 451	3 305 661	2 682 207	3 738 837	6 611 395	9 034 752
**Law enforcement (€)**	**54 643 968 (49 212 651–62 093 800)**	**70 330 431 (63 440 101–79 528 082)**	**75 726 925 (68 058 399–85 658 149)**	**70 337 433 (61 648 481–78 726 452)**	**83 912 140 (75 838 949–95 414 842)**	**85 521 702 (75 660 431–95 888 963)**
Traffic accidents	18 189 374 (16 381 448–20 669 205)	21 700 091 (19 574 115–24 537 978)	16 990 072 (15 269 564–19 218 238)	20 617 166 (18 070 278–23 076 139)	21 468 588 (19 403 094–24 411 509)	27 050 991 (23 931 816–30 330 213)
Imprisonment	7 889 260 (7 105 109–8 964 834)	11 453 429 (10 331 327–12 951 282)	11 868 492 (10 666 623–13 424 988)	12 469 219 (10 928 866–13 956 400)	14 211 096 (12 843 846–16 159 157)	12 828 297 (11 349 102–14 383 392)
Pre-trial investigations	10 521 625 (9 475 832–11 956 080)	8 319 744 (7 504 652–9 407 781)	10 617 559 (9 542 366–12 010 001)	11 967 523 (10 489 146–13 394 869)	16 486 983 (14 900 769–18 747 024)	15 484 624 (13 699 134–17 361 728)
Financial loss due to criminal offences	8 211 421 (7 395 250–9 330 917)	19 643 911 (17 719 381–22 212 896)	25 056 094 (22 518 776–28 342 082)	10 977 338 (9 621 281–12 286 585)	14 499 822 (13 104 793–16 487 462)	9 697 321 (8 579 149–10 872 866)
Court system	9 832 288 (8 855 011–11 172 763)	9 213 256 (8 310 626–10 418 144)	11 194 708 (10 061 070–12 662 840)	14 306 186 (12 538 909–16 012 460)	17 245 651 (15 586 446–19 609 691)	20 460 469 (18 101 229–22 940 763)
**Indirect costs**						
**Productivity loss (€)**	**366 535 636 (344 021 890–392 561 216)**	**359 117 137 (333 772 957–379 460 639)**	**328 950 079 (302 954 634–347 895 986)**	**333 187 702 (312 740 240–356 799 429)**	**351 844 266 (321 314 119–369 015 447)**	**385 567 773 (362 882 201–411 725 516)**
Male	308 239 995 (286 859 654–332 379 063)	306 364 161 (281 553 117–325 882 889)	280 139 914 (255 478 051–297 968 243)	283 405 653 (262 521 552–305 790 191)	298 833 683 (269 424 404–313 897 627)	325 997 452 (305 098 648–350 249 679)
Female	58 295 641 (50 470 151–66 496 069)	52 752 976 (45 034 171–60 929 980)	48 810 165 (41 187 467–56 320 841)	49 782 049 (42 702 499–57 605 360)	53 010 583 (45 692 510–60 311 207)	59 570 320 (50 904 368–68 756 963)
**Total alcohol-attributable costs (€)**	**536 305 171 (512 434 726–568 408 233)**	**540 371 454 (511 563 694–568 227 235)**	**516 825 382 (487 247 350–542 275 206)**	**516 615 771 (490 644 249–548 863 578)**	**556 865 100 (522 595 473–582 048 198)**	**590 765 543 (562 753 299–623 752 984)**
**Alcohol-attributable % of GDP (%)**	**1.29%**	**1.26%**	**1.15%**	**1.10%**	**1.12%**	**1.18%**

Group I: fully alcohol-attributable causes; Group II: partially alcohol-attributable causes; Group III: external causes.

### Healthcare costs

On average 10% of the healthcare costs were attributable to the disease group I (fully alcohol-attributable causes). Meanwhile, external causes (disease group III) amounted to 40% of healthcare costs. The largest healthcare cost component (50%) was associated with partially alcohol-attributable disease group II, where the costs were mainly driven by circulatory system diseases and cancer. On average, the alcohol-attributable healthcare costs amounted to 16.3% of the annual total healthcare expenditure.

### Childcare costs

Table 1 shows that the total alcohol-related costs of childcare in 2015 were €59.61 million, of which €53.14 million were spent within child guardianship, and €58.88 and €45.12 million in 2020, respectively. Alcohol-related social benefits costs account for the lowest share of the total costs, from €3.37 million in 2015 to €4.72 million in 2020. During the period analysed, the cost of professionals who worked with families with problematic drinking increased from €3.1 million in 2015 to €9 million in 2020. Over the 6 years analysed, the alcohol-related childcare costs, on average, were around €58.31 million per year and remained relatively stable, except for 2016, when the costs were €54.96 million, and €60.65 million in 2017.

### Law enforcement and justice system costs

Between 2015 and 2020, the Lithuanian law enforcement system registered on average 10 133 crimes annually committed by alcohol-intoxicated persons and 293 road traffic accidents caused by driving under the influence of alcohol (see Tables S4 and S5). This represented approximately 17.3% of all crimes committed during the analysed period. Law enforcement and justice system costs on average amounted to €73.41 million annually. The cost of property damage due to alcohol-impaired driving accounted on average for 29% of all law enforcement costs or approximately €21 million per year. The rest of the analysed costs were distributed fairly evenly amongst other categories with financial loss due to criminal offences committed accounting for 20% or €14.68 million, alcohol-attributable costs incurred in the court system accounting for 19% or €13.71 million, alcohol-attributable cost of pre-trial investigations accounting for 17% or €12.23 million, and cost of imprisonment accounting for 16% or €11.79 million per year.

### Cost of productivity loss due to premature mortality

With respect to the costs of lost productivity, there was an average of 1 967 premature deaths annually, or 31 113 potential YLLs, due to alcohol between 2015 and 2020. The number of premature deaths was 5.4 times higher among Lithuanian males than females, 84.3% and 15.7% respectively. Lost productivity contributed the largest amount to the economic costs of alcohol use, at approximately €354.20 million annually. The analysis by sex showed that the 85% of indirect costs were generated by males (a difference of €246.79 million). On average, annual lost productivity costs were €300.50 million among males and €53.70 million among females.

## Discussion

Alcohol use has a considerable negative impact on the economic prosperity of Lithuania given that a conservative estimate of the economic cost was equivalent to about 1.18% of annual GDP. These findings are similar to previous studies conducted in high-income countries and now are closer to the Western Europe average of 1.3% (0.9%–1.7%), than the Eastern European average of 2.1% (0.7%–3.4%) [2]. Indirect costs in the form or productivity losses made up the largest cost component accounting for approximately 65% of the total costs. The distribution between direct and indirect costs was very close to the global average of 61.2% (55.0%–71.4%) [2].

Alcohol *per capita* (APC) consumption in Lithuania among the population 15 years old and older has been among the highest globally in recent decades [29–31]. During the study period, the APC ranged from 14.5 litres of pure alcohol in 2015 to 11.4 litres in 2020 [32]. Although alcohol consumption trends continue to decline with the exception of the COVID-19 period (APC was down to 11.0 litres in 2023 [32]), alcohol use remains a significant health risk factor in Lithuania with a significant footprint on alcohol-attributable costs.

We observed a decline in alcohol-attributable costs in 2017 and 2018, but there was an increase in the 2019–20 period. This may be due to multiple reasons, such as the implementation of alcohol control policies in 2017 and 2018 [5] (primarily due to the reduction in alcohol-attributable mortality during those 2 years [3, 33], among others. In terms of the fluctuation of alcohol-related childcare costs during the analysed period, this was likely to deinstitutionalization of childcare and child rights protection reforms, which in 2017 resulted in the doubling of childcare benefits for families (from €152 in 2015 to €304 in 2017). This led to an increase in alcohol-related costs. In 2018, the child rights protection reform took effect, which increased services for families fostering children and reduced annual numbers of children entering the care system. In terms of law enforcement costs, broader socioeconomic factors such as rising salaries for law enforcement and justice system officers, increased incarceration costs, or more in-depth pre-trial investigation procedures may have driven up per case unit costs, especially in later years of the analysis.

We likely underestimated alcohol-attributable costs for several reasons. First, there were costs that we were unable to include in this analysis, including but not limited to productivity losses caused by alcohol-attributable hospitalizations, unemployment, absence of work, presenteeism and hangover due to alcohol, paid state social insurance sickness benefits, pensions for work incapacity, and state pensions for disability group.

Second, the provision of social services is within the mandate of the municipalities. In our calculations, only the mandatory expenditure for child guardianship was considered. While most municipalities allocate higher amounts, there is a lack of collected data. The costs of psychologists, addiction specialists, and court proceedings related to child guardianship and parental rights restrictions are not included in the calculations of alcohol-related childcare costs. On the other hand, the range of services has expanded after the child rights protection reform in 2018, which is reflected in the costs of remuneration. Therefore, we could not draw any association between the change in costs and the change in alcohol-attributable costs related to child-care services in this period.

Third, due to a lack of available data, law enforcement costs do not cover the cost of arrests or corrections. Additionally, no estimates are available on the costs of policing due to alcohol (i.e. police officers responding to calls by drunk people or crimes committed by drunk people) and impacts of illicit trade, including costs related to contraband, smuggling, or counterfeit alcohol.

Fourth, we did not include the costs of research and prevention, although some fragmented data exists. Between 2016 and 2020, the State Public Health Promotion Fund alone allocated approximately €1.68 million to alcohol prevention and research projects and media campaigns [34]. The economic costs would have been higher if these omitted factors could have been incorporated into the analysis.

Lastly, due to methodological limitations, we omitted intangible costs (i.e. costs due to pain, suffering, and bereavement).

On the other hand, we used the human capital approach, which assumes that the time of premature deaths until retirement is an economic loss because it assumes full employment and no replacement of the deceased possibilities. Obviously, these assumptions are wrong, as most positions will be replaced [7], so some economists argue that the human capital approach leads to an overestimate.

While we used annual data from 2015 to 2020 for all cost components, there is no available evidence or previous research specifically validating the accuracy of the administrative data sources used in this study. As with most secondary data, it is important to acknowledge that the collected data are unlikely to be 100% complete even though it represents official data. For example, not all crimes are reported or recorded by law enforcement agencies, and not all alcohol-attributable diseases are captured or reimbursed as per the records of health insurance fund data.

Even based on our overall conservative estimate of the alcohol-attributable economic burden in Lithuania between 2015 and 2020, the estimated costs were 1.74 times higher than alcohol excise tax revenues (adjusted for 2020) which ranged from €292 million in 2015 to €384 million in 2020 (adjusted to costs of 2020). This study attempted to provide detailed estimates of the alcohol-attributable economic costs in Lithuania. It is hoped that it will stimulate further work in the area beyond alcohol use. However, even now it is clear that alcohol use has remained a major cost factor despite the successful major alcohol control policy implementations of 2008–9 and 2017–18 [3, 35]. The success of the alcohol policies in 2017–18 can be seen in Table 1, when the alcohol-attributable economic cost dropped substantially, both in value and relative to the GDP (see also [3, 33]). More continuous alcohol control policy is needed if Lithuania wants to further reduce alcohol-attributable burden. In addition to making sure that alcohol does not become more affordable due to increases in disposable income, specific efforts should be directed to change patterns of male drinking in Lithuania, which are still characterized by heavy episodic drinking occasions. Changing this drinking pattern via screening and brief interventions, specific fiscal policy measures and further restricting on-premises hours should be a priority [36].

## Supplementary data

Supplementary data are available at *EURPUB* online.

Conflict of interest: None declared.

## Supplementary Material

ckaf069_Supplementary_Data

## Data Availability

The data underlying this article will be shared on reasonable request to the corresponding author.
Key points Despite a positive trend of decreasing alcohol consumption, alcohol continues to impose a substantial economic burden in Lithuania. A considerable proportion of the economic cost of alcohol was due to premature mortality. Our study shows females incur fewer direct and indirect costs, attributable to lower drinking prevalence and less detrimental drinking patterns. Alcohol control policies, in particular excise taxation increases and availability restrictions, may be associated with the decrease of this burden.
