# Supplementary material for: The economic costs of alcohol consumption in Lithuania, 2015–20
Source: Eur J Public Health. 2025 May 9;35(4):726–32. doi: 10.1093/eurpub/ckaf069 (PMC12311340; doi:10.1093/eurpub/ckaf069)
Supplement: ckaf069_Supplementary_Data [file ckaf069_supplementary_data.docx]

# Appendices

**Table S1**: Consumer price index (CPI) for conversion factor to 2020, 2021, 2022 or 2023 Euros

| **CPI** | **2015** | **2016** | **2017** | **2018** | **2019** | **2020** |
| --- | --- | --- | --- | --- | --- | --- |
| **2020** | 1.1124910000 | 1.1025678890 | 1.0632284368 | 1.0352759766 | 1.0120003748 | 1.0000000000 |
| **2021** | 1.1647780000 | 1.1543885035 | 1.1132000998 | 1.0839338758 | 1.0595643224 | 1.0469999308 |
| **2022** | 1.3942390000 | 1.3818027750 | 1.3325002652 | 1.2974686018 | 1.2682982519 | 1.2532586780 |
| **2023** | 1.4270040000 | 1.4142755203 | 1.3638143880 | 1.3279594708 | 1.2981036097 | 1.2827106017 |

Source: CPI – Statistics Lithuania (8); authors’ calculations.

**Table S2:** Average Alcohol-Attributable Fractions for the Estimates of Alcohol-Attributable Healthcare Costs, 2015-2020.

| **Disease (ICD-10 codes)** | **2015** | **2016** | **2017** | **2018** | **2019** | **2020** |
| --- | --- | --- | --- | --- | --- | --- |
| **Group I. Conditions fully attributable to alcohol** | | | | | | |
| Diseases and conditions: E24.4, F10, G31.2, G62.1, G72.1, I42.6, K29.2, K70, K85.2, K86.0, O35.4, R78.0, T51.0, T51.9 | 1.000  (1.000-1.000) | 1.000  (1.000-1.000) | 1.000  (1.000-1.000) | 1.000  (1.000-1.000) | 1.000  (1.000-1.000) | 1.000  (1.000-1.000) |
| **Group II. Conditions partially attributable to alcohol** | | | | | | |
| Tuberculosis (A15-A19, B90) | 0.299  (0.195-0.435) | 0.29  (0.199-0.426) | 0.272  (0.164-0.375) | 0.263  (0.189-0.396) | 0.265  (0.181-0.387) | 0.271  (0.18-0.391) |
| HIV/AIDS (B20–B24) | 0.052  (0.039-0.064) | 0.049  (0.036-0.062) | 0.044  (0.032-0.057) | 0.042  (0.03-0.054) | 0.042  (0.031-0.054) | 0.044  (0.032-0.056) |
| Lower Respiratory Infections: Pneumonia (J09–J22, J85, P23, U04) | 0.181  (0.16-0.215) | 0.176  (0.15-0.207) | 0.167  (0.148-0.197) | 0.162  (0.135-0.186) | 0.163  (0.122-0.173) | 0.167  (0.145-0.196) |
| Lip And Oral Cavity Cancer (C00–C08) | 0.441  (0.378-0.496) | 0.455  (0.391-0.506) | 0.461  (0.401-0.513) | 0.461  (0.404-0.51) | 0.449  (0.39-0.498) | 0.434  (0.371-0.491) |
| Other Pharynx Cancer (C09–C14) | 0.441  (0.378-0.496) | 0.455  (0.391-0.506) | 0.461  (0.401-0.513) | 0.461  (0.404-0.51) | 0.449  (0.39-0.498) | 0.434  (0.371-0.491) |
| Oesophagus cancer (C15) | 0.272  (0.228-0.311) | 0.281  (0.236-0.322) | 0.286  (0.243-0.327) | 0.286  (0.239-0.325) | 0.278  (0.23-0.317) | 0.267  (0.22-0.308) |
| Colon and rectum cancer (C18-C21) | 0.175  (0.137-0.21) | 0.18  (0.142-0.214) | 0.182  (0.145-0.218) | 0.182  (0.144-0.216) | 0.178  (0.141-0.213) | 0.174  (0.136-0.209) |
| Liver cancer (C22) | 0.249  (0.206-0.287) | 0.251  (0.208-0.29) | 0.252  (0.209-0.292) | 0.253  (0.21-0.293) | 0.251  (0.209-0.29) | 0.252  (0.207-0.291) |
| Female Breast Cancer (C50) | 0.055  (0.04-0.072) | 0.059  (0.043-0.076) | 0.06  (0.042-0.08) | 0.06  (0.044-0.079) | 0.057  (0.041-0.075) | 0.053  (0.039-0.071) |
| Larynx cancer (C32) | 0.278  (0.211-0.336) | 0.287  (0.22-0.347) | 0.292  (0.225-0.35) | 0.292  (0.22-0.348) | 0.284  (0.213-0.341) | 0.274  (0.203-0.331) |
| Hypertensive heart disease (I10–I15) | 0.122  (0.102-0.151) | 0.119  (0.099-0.149) | 0.114  (0.094-0.143) | 0.111  (0.091-0.141) | 0.112  (0.091-0.141) | 0.114  (0.094-0.143) |
| Ischaemic heart disease (I20–I25) | 0.166  (0.129-0.212) | 0.163  (0.117-0.203) | 0.158  (0.115-0.198) | 0.156  (0.117-0.195) | 0.157  (0.11-0.194) | 0.159  (0.12-0.199) |
| Ischaemic stroke (G45, G46, I63–I66.9, I67.2-I67.8, I69.3, I69.4) | -0.022  (-0.051-0.059) | -0.028  (-0.061-0.049) | -0.037  (-0.075-0.029) | -0.041  (-0.059-0.04) | -0.04  (-0.079-0.019) | -0.038  (-0.077-0.03) |
| Haemorrhagic and other non-ischaemic stroke (I60–I62, I67.0, I67.1, I69.0, I69.1, I69.2) | 0.181  (0.132-0.251) | 0.177  (0.12-0.234) | 0.17  (0.107-0.219) | 0.166  (0.111-0.219) | 0.167  (0.107-0.217) | 0.17  (0.123-0.232) |
| Atrial fibrillation and flutter (I48) | 0.127  (0.101-0.154) | 0.123  (0.098-0.148) | 0.116  (0.09-0.139) | 0.112  (0.088-0.134) | 0.113  (0.089-0.136) | 0.115  (0.09-0.138) |
| Diabetes mellitus (E11–E14) | -0.029  (-0.064-0.008) | -0.029  (-0.064-0.007) | -0.027  (-0.059-0.006) | -0.026  (-0.06-0.007) | -0.026  (-0.059-0.006) | -0.027  (-0.061-0.007) |
| Epilepsy (G40, G41) | 0.252  (0.208-0.296) | 0.245  (0.2-0.288) | 0.231  (0.188-0.275) | 0.224  (0.183-0.266) | 0.225  (0.184-0.268) | 0.23  (0.188-0.275) |
| Liver cirrhosis and liver disease (K71-K76) | 0.613  (0.559-0.681) | 0.605  (0.538-0.666) | 0.587  (0.512-0.64) | 0.578  (0.521-0.645) | 0.581  (0.514-0.642) | 0.587  (0.514-0.64) |
| **Group III. External causes partially attributable to alcohol** | | | | | | |
| Injuries (S00-T98) | 0.329  (0.297-0.374) | 0.327  (0.295-0.369) | 0.318  (0.286-0.359) | 0.314  (0.275-0.352) | 0.315  (0.284-0.358) | 0.317  (0.28-0.355) |

Source: Shield et al (2020) (12) and Rehm et al. (2024) (3); authors’ calculations.

**Table S3:** Alcohol-Attributable Fractions for the Estimates of Alcohol-Attributable Lost Productivity Costs, 2020.

| **Disease (ICD-10 codes)** | **Age Group** | | | | | |
| --- | --- | --- | --- | --- | --- | --- |
|  | **15-34** | | **35-64** | | **65+** | |
|  | **Male** | **Female** | **Male** | **Female** | **Male** | **Female** |
| **Group I. Conditions fully attributable to alcohol** | | | | | | |
| Diseases and conditions: E24.4, F10, G31.2, G62.1, G72.1, I42.6, K29.2, K70, K85.2, K86.0, O35.4, R78.0, T51.0, T51.9 | 1.000  (1.000-1.000) | 1.000  (1.000-1.000) | 1.000  (1.000-1.000) | 1.000  (1.000-1.000) | 1.000  (1.000-1.000) | 1.000  (1.000-1.000) |
| **Group II. Conditions partially attributable to alcohol** | | | | | | |
| Tuberculosis (A15-A19, B90) | 0.376  (0.349-0.454) | 0.241  (0.048-0.524) | 0.408  (0.28-0.407) | 0.223  (0.045-0.488) | 0.319  (0.193-0.364) | 0.125  (0.023-0.299) |
| HIV/AIDS (B20–B24) | 0.077  (0.047-0.107) | 0.025  (0.01-0.041) | 0.091  (0.059-0.122) | 0.022  (0.009-0.037) | 0.059  (0.025-0.088) | 0.007  (0.001-0.019) |
| Lower Respiratory Infections: Pneumonia (J09–J22, J85, P23, U04) | 0.321  (0.27-0.344) | 0.057  (0.012-0.113) | 0.351  (0.282-0.391) | 0.053  (0.012-0.105) | 0.268  (0.204-0.356) | 0.03  (0.006-0.063) |
| Lip And Oral Cavity Cancer (C00–C08) | 0.608  (0.52-0.676) | 0.344  (0.255-0.422) | 0.653  (0.572-0.717) | 0.332  (0.222-0.423) | 0.564  (0.43-0.649) | 0.219  (0.075-0.339) |
| Other Pharynx Cancer (C09–C14) | 0.608  (0.52-0.676) | 0.344  (0.255-0.422) | 0.653  (0.572-0.717) | 0.332  (0.222-0.423) | 0.564  (0.43-0.649) | 0.219  (0.075-0.339) |
| Oesophagus cancer (C15) | 0.386  (0.325-0.431) | 0.195  (0.133-0.252) | 0.429  (0.366-0.473) | 0.188  (0.117-0.252) | 0.351  (0.242-0.416) | 0.129  (0.017-0.223) |
| Colon and rectum cancer (C18-C21) | 0.27  (0.216-0.321) | 0.094  (0.046-0.142) | 0.295  (0.236-0.351) | 0.091  (0.037-0.14) | 0.32  (0.27-0.362) | 0.058  (-0.019-0.126) |
| Liver cancer (C22) | 0.203  (0.144-0.259) | 0.23  (0.175-0.279) | 0.219  (0.153-0.278) | 0.244  (0.188-0.295) | 0.276  (0.197-0.34) | 0.325  (0.249-0.389) |
| Female Breast Cancer (C50) | 0  (0-0) | 0.121  (0.082-0.167) | 0  (0-0) | 0.115  (0.076-0.16) | 0  (0-0) | 0.063  (0.036-0.1) |
| Larynx cancer (C32) | 0.389  (0.299-0.476) | 0.204  (0.123-0.275) | 0.43  (0.336-0.514) | 0.198  (0.11-0.28) | 0.355  (0.229-0.458) | 0.139  (-0.002-0.252) |
| Hypertensive heart disease (I10–I15) | 0.19  (0.139-0.234) | 0.068  (0.046-0.115) | 0.203  (0.152-0.249) | 0.062  (0.044-0.11) | 0.165  (0.104-0.215) | 0.035  (0.025-0.07) |
| Ischaemic heart disease (I20–I25) | 0.263  (0.238-0.303) | 0.043  (-0.128-0.175) | 0.285  (0.271-0.331) | 0.051  (-0.078-0.164) | 0.243  (0.184-0.271) | 0.112  (0.011-0.19) |
| Ischaemic stroke (G45, G46, I63–I66.9, I67.2-I67.8, I69.3, I69.4) | 0.088  (0.079-0.091) | -0.151  (-0.283-0.178) | 0.093  (0.087-0.106) | -0.158  (-0.276--0.028) | 0.073  (0.024-0.049) | -0.11  (-0.241-0.033) |
| Haemorrhagic and other non-ischaemic stroke (I60–I62, I67.0, I67.1, I69.0, I69.1, I69.2) | 0.118  (0.093-0.156) | 0.227  (0.124-0.343) | 0.126  (0.118-0.183) | 0.218  (0.115-0.33) | 0.141  (0.11-0.235) | 0.178  (0.071-0.269) |
| Atrial fibrillation and flutter (I48) | 0.184  (0.127-0.237) | 0.078  (0.053-0.109) | 0.204  (0.146-0.261) | 0.072  (0.046-0.101) | 0.151  (0.091-0.201) | 0.04  (0.022-0.062) |
| Diabetes mellitus (E11–E14) | 0.049  (0.005-0.091) | -0.123  (-0.172--0.067) | 0.054  (0.005-0.102) | -0.115  (-0.164--0.06) | 0.065  (-0.002-0.125) | -0.053  (-0.106--0.002) |
| Epilepsy (G40, G41) | 0.361  (0.261-0.456) | 0.161  (0.105-0.222) | 0.394  (0.302-0.485) | 0.149  (0.098-0.209) | 0.305  (0.196-0.404) | 0.085  (0.045-0.13) |
| Liver cirrhosis and liver disease (K71-K76) | 0.489  (0.382-0.513) | 0.695  (0.551-0.816) | 0.528  (0.461-0.577) | 0.679  (0.538-0.799) | 0.519  (0.409-0.567) | 0.593  (0.463-0.715) |
| **Group III. External causes partially attributable to alcohol** | | | | | | |
| Injuries and External Causes (V00-Y98) | 0.554  (0.505-0.576) | 0.205  (0.111-0.313) | 0.582  (0.538-0.597) | 0.147  (0.081-0.229) | 0.495  (0.432-0.543) | 0.059 (0.031-0.095) |

Source: Rehm et al. (2024) (3) and Miščikienė et al (2024) (13); authors’ calculations.

**Table S4**: Proportion of Alcohol-Attributable Criminal Offences, 2015–2020.

|  | **2015** | **2016** | **2017** | **2018** | **2019** | **2020** |
| --- | --- | --- | --- | --- | --- | --- |
| **Total number of criminal offences** | 72 343 | 59 075 | 63 846 | 57 830 | 51 449 | 46 306 |
| **Criminal offences committed by alcohol intoxicated persons** | 10 364 | 7 713 | 9 780 | 10 787 | 10 797 | 11 356 |
| **The proportion attributable to alcohol use (%)** | **14.33** | **13.06** | **15.32** | **18.65** | **20.99** | **24.52** |

Source: Information Technology and Communications Department under the Ministry of the Interior of the Republic of Lithuania (14); authors’ calculations.

**Table S5**: Proportion of Alcohol-Attributable Traffic Accidents, 2015–2020.

|  | **2015** | **2016** | **2017** | **2018** | **2019** | **2020** |
| --- | --- | --- | --- | --- | --- | --- |
| **Total number of road traffic accidents with injuries** | 3 033 | 3 201 | 3 052 | 2 925 | 3 188 | 2 788 |
| **Traffic Accidents Involving Intoxicated Participants** | 315 | 351 | 245 | 267 | 270 | 308 |
| **The proportion attributable to alcohol use (%)** | **10.39** | **10.97** | **8.03** | **9.13** | **8.47** | **11.05** |

Source: Police Department (15); authors’ calculations.
